# Socioeconomic Status, Culture, and Reading Comprehension in Immigrant Students

**DOI:** 10.3389/fpsyg.2021.752273

**Published:** 2021-11-19

**Authors:** Joaquín A. Ibáñez-Alfonso, Juan Andrés Hernández-Cabrera, Jon Andoni Duñabeitia, Adelina Estévez, Pedro Macizo, María Teresa Bajo, Luis J. Fuentes, David Saldaña

**Affiliations:** ^1^Individual Differences, Language and Cognition Lab, Department of Developmental and Educational Psychology, Universidad de Sevilla, Seville, Spain; ^2^Human Neuroscience Lab, Department of Psychology, Universidad Loyola Andalucía, Seville, Spain; ^3^Department of Psychobiology and Methodology of Behavioral Sciences, Universidad de La Laguna, Santa Cruz de Tenerife, Spain; ^4^Centro de Investigación Nebrija en Cognición, Universidad Antonio de Nebrija, Madrid, Spain; ^5^Department of Language and Culture, The Arctic University of Norway, Tromsø, Norway; ^6^Department of Cognitive, Social and Organizational Psychology, Universidad de La Laguna, Santa Cruz de Tenerife, Spain; ^7^Memory and Language Research Group, Department of Experimental Psychology, Universidad de Granada, Granada, Spain; ^8^Department of Basic Psychology and Methodology, Universidad de Murcia, Murcia, Spain

**Keywords:** reading comprehension, immigrants, socioeconomic status, second language learners, culture, Spanish

## Abstract

Research on reading comprehension in immigrant students is heterogeneous and conflicting. Differences in socioeconomic status and cultural origins are very likely confounds in determining whether differences to native pupils can be attributed to immigrant status. We collected data on 312 Spanish students of Native, of Hispanic origin–therefore with the same family language as native students- and Non-Hispanic origin, while controlling for socioeconomic status, non-verbal reasoning and school membership. We measured reading comprehension, knowledge of syntax, sentence comprehension monitoring, and vocabulary. Differences among groups appeared only in vocabulary and syntax (with poorer performance in the non-Hispanic group), with no differences in reading comprehension. However, regression analyses showed that most of the variability in reading comprehension was predicted by age, socioeconomic status, non-verbal reasoning, and comprehension monitoring. Group membership did not significantly contribute to explain reading comprehension variability. The present study supports the idea that socioeconomically disadvantaged students, both native and immigrants from diverse cultural backgrounds, irrespective of the language of origin, are probably equally at risk of poor reading comprehension.

## Introduction

An estimated 11% of children in schools worldwide are born to immigrant parents and can be considered to have an immigrant background (IB). This figure hides a great deal of variability among countries: Numbers range from under 1% for some (e.g., Korea, Poland or Japan) to nearly 30% for others (e.g., Luxembourg or Macao-China), and in Organisation for Economic Co-operation and Development (OECD) and European countries around 25% 15-year-old students was foreign-born or had at least one immigrant parent. In all of them it is common for IB children to underachieve academically, especially first-generation immigrant students (OECD, 2015, 2018). In Spain, 11.5% of primary and secondary education pupils are of immigrant background (Ministerio de Educación y Formación Profesional, 2021). One of the largest groups of IB children is of Hispanic origin, representing around 30% of the IB children. Different studies have shown a poorer academic performance of these students of Hispanic origin, despite sharing the same language with their Spanish native peers (Ministerio de Educación de España, 2010, 2011; OECD, 2014).

This underperformance could be related to poor reading abilities, among other core academic abilities such as mathematics and science (OECD, 2018). The Simple View of Reading (SVR) proposes that reading is the result of the multiplicative interaction between the ability to decode written text and comprehend oral language (Hoover and Gough, 1990). The SVR model was initially founded on English. Although the influence of decoding and linguistic comprehension over reading comprehension may vary in different languages and orthographies, several studies have found that it is applicable to a wide range of writing systems (e.g., Florit and Cain, 2011; Joshi et al., 2012). Either of these necessary skills could be problematic in the case of IB children (Mancilla-Martinez et al., 2011), many of whom have a primary language (L1) different from the one used at school (L2). Lack of familiarity with the language could affect decoding, as these children may have less specified phonological representations for their L2, or irregular knowledge of letter-sound correspondences. Limited oral language proficiency could also affect their reading comprehension, via poor vocabulary or syntax.

Interestingly, decoding does not appear to be problematic. Most studies find similar levels of word reading between these children and their native peers (e.g., Hutchinson et al., 2004; Chiappe and Siegel, 2006; Jongejan et al., 2007; Leikin et al., 2010; Lervåg and Aukrust, 2010; Netten et al., 2011; Geva and Farnia, 2012; Lipka and Siegel, 2012; Verhoeven and van Leeuwe, 2012). Only a few of them find better performance of native students (e.g., Verhoeven and Vermeer, 2006; Proctor et al., 2012; Shany and Geva, 2012). However, existing research on reading comprehension is much less conclusive. Studies that find differences, always found them in favor of native pupils (e.g., Lervåg and Aukrust, 2010; Burgoyne et al., 2011; Netten et al., 2011; Geva and Farnia, 2012; Pasquarella et al., 2012; Proctor et al., 2012; Shany and Geva, 2012; Verhoeven and van Leeuwe, 2012; Melby-Lervåg and Lervåg, 2014; Kigel et al., 2015). But there are a large number of studies in which no significant differences are found (e.g., Lesaux and Siegel, 2003; Lesaux et al., 2007; Lipka and Siegel, 2007, 2012; Leikin et al., 2010; Rodríguez-Parra et al., 2012). In an example of the complexity of the issue, in a study with German children differences were found for one group of immigrant pupils (Turkish), but not for others (Marx et al., 2015).

There are many potential reasons for this variability in the literature. One is IB students’ primary language. Structural similarities between L1 and L2 may affect the extent to which their performance differs. Some IB children may share the L1 of their native peers. For example, children from the Commonwealth emigrating or born to immigrant parents in the United States or the United Kingdom do not have to overcome a language barrier as great as the one facing non-English-speaking children. A similar situation can be found in Spain with IB children coming from Spanish-speaking countries. The L1 most spoken among IB children of Spain is Spanish (27% of the total), mainly coming from Central and South America. Although the biggest groups of IB children in Spain are those coming from Europe (30.9%) and Africa (30.7%), they have a wide range of L1s, different from native students (Ministerio de Educación y Formación Profesional, 2021). Additionally, the Spanish school system shows some peculiarities regarding the presence of bilingualism in different autonomous communities. For example, in the autonomous communities of Catalonia, Basque Country, or Galicia, students are usually bilingual in Spanish and the co-official languages of these regions. However, most studies treat IB children as a group or only focus on children learning an L2. A mixed proportion of L1 and L2 children among IB participants could partially account for the conflicting findings of studies looking at reading comprehension. In the few studies in which different L1 immigrant groups are analyzed separately, smaller differences were found in reading comprehension between these same-L1 and native children, than between different-L1 and native groups (Navarro and Huguet, 2005; Verhoeven and Vermeer, 2006; Proctor et al., 2012).

In addition to native language, socioeconomic status (SES) is a potential confound. On occasions, researchers have not taken this variable into consideration (e.g., Hutchinson et al., 2004; Navarro and Huguet, 2005; Burgoyne et al., 2011; Netten et al., 2011; Proctor et al., 2012). Other studies attempt to control SES by sampling participants of a wide range of SES (e.g., Lesaux et al., 2007; Lipka and Siegel, 2007, 2012) or including a proxy variable for SES such as home neighborhood or schools (e.g., Droop and Verhoeven, 2003; Verhoeven and Vermeer, 2006; Lervåg and Aukrust, 2010; Geva and Farnia, 2012; Pasquarella et al., 2012; Verhoeven and van Leeuwe, 2012). In some cases, several indicators of SES have been analyzed, such as housing density, parental occupation, or poverty indicators, but not used as covariates in performance analyses (e.g., Shany and Geva, 2012). Finally, most of the studies that did statistically control the influence of SES on outcome measures found no significant differences in reading comprehension between IB and native children (e.g., Lesaux and Siegel, 2003; Leikin et al., 2010; Rodríguez-Parra et al., 2012). A notable exception are the analyses of the Programme for International Student Assessment (PISA) studies (OECD, 2014), that show differences between native and IB children after accounting for the influence of SES. According to data from the Ministry of Education (Consejo Escolar del Estado, 2019), the distribution of immigrants under 16 years of age is uneven throughout the Spanish territory. When we look at the percentage of foreign population with respect to the total population of each region, the Balearic Islands (15.2%), Melilla (15.2%), and the Region of Murcia (14.2%) stand out as the communities with the highest relative percentage IB children in their schools. Territorial inequality can also be found in other parameters such as the level of studies of adult population, a value that positively correlates with the academic performance of the student population. In Spain, 66.2% of the population between 25 and 34 years old has post-compulsory studies, this percentage varying between the 80.7% registered in the Basque Country, and the 56.1% registered in Andalusia. The European average in this measure is 83.4%. Data on the economic, social and cultural level of families (ESCS index, average = 0, *SD* = 1) indicate that the communities of Madrid (−0.10) and the Basque Country (−0.25) would be the only ones that would show values close to the OECD average, while the Canary Islands (−0.80), the Region of Murcia (−0.82) and Andalusia (−0.87) would be the furthest from this average (Instituto Nacional de Evaluación Educativa, 2016).

Reading comprehension can be affected by limited decoding skills or oral language comprehension (Hoover and Gough, 1990). Since immigrant readers’ decoding seems to follow the levels of native children, we shall be focusing on the components more closely related to language comprehension. Vocabulary is one of the more relevant predictors of reading comprehension (Nation, 2005; Perfetti and Landi, 2005). It is inevitably associated with the prior cultural and linguistic knowledge that IB children have of both their native language and the language in which they learn to read. Any lexical limitations will impact negatively on IB children’s L2 reading comprehension (Verhoeven and van Leeuwe, 2012; Silverman et al., 2015). Syntactic knowledge and awareness are also related to comprehension. Children’s poor syntactic awareness has been systematically associated with low performance in L1 and L2 reading comprehension tasks (e.g., Yuill and Oakhill, 1991; Nation, 2005; Perfetti and Landi, 2005; Lesaux et al., 2007; Verhoeven and van Leeuwe, 2012; Bellocchi et al., 2017). IB children generally underperform with respect to their native peers on these variables (e.g., Droop and Verhoeven, 2003; Navarro and Huguet, 2005; Verhoeven and Vermeer, 2006; Lipka and Siegel, 2007, 2012; Lervåg and Aukrust, 2010; Geva and Farnia, 2012; Proctor et al., 2012; Shany and Geva, 2012).

It has been argued that certain cognitive factors other than oral language and decoding skill need to be added to the SVR model to adequately capture individual differences in reading (Pennington and Bishop, 2009). Executive functions, such as working memory, inhibition, planning, or monitoring are some of these factors. They have been found to explain additional variance in individual differences on reading comprehension, once word reading and oral language have been taken into consideration (e.g., Christopher et al., 2012; Georgiou and Das, 2016; Potocki et al., 2017; Follmer, 2018; Nouwens et al., 2021). In this context, working memory, inhibition, and comprehension monitoring have been found to correlate with L2 learners’ reading comprehension (Cain and Oakhill, 1999; Raudszus et al., 2018).

The influence of all these cognitive and linguistic components on the reading of immigrant children, and their relative importance in explaining individual differences are of interest. Various studies have analyzed the similarity of L1 and L2 models of reading (Verhoeven and van Leeuwe, 2008, 2012; Chen et al., 2012; Lipka and Siegel, 2012). It seems that component skills could evolve differently in IB and native children with age. From approximately the middle grades of Primary school (3rd–5th), word reading and decoding seem to lose weight developing roughly at the same rate in L1 and L2. For example, Verhoeven and van Leeuwe (2012), conducted a longitudinal study with 1293 L1 and 394 L2 learners of Dutch to assess the SVR model throughout the primary grades. They found in the longitudinal analysis that the SVR model showed the same validity for L1 and L2 learners, following an equivalent pattern, with a decreasing impact of word decoding with time, while the impact of listening comprehension on reading comprehension increased through primary grades. Lesaux and Siegel (2003), found that early interventions beginning in kindergarten can even foster L2 bilinguals outperforming L1’s word decoding skills at grade 2. Nevertheless, although word decoding performance can be equivalent in both groups, L2 tends to underperform in listening and reading comprehension at the end of primary grades (Verhoeven and van Leeuwe, 2012). The relative importance of comprehension grows from there supported by vocabulary, a knowledge that is usually affected by differences in the SES of the children, low-SES showing a lower performance (e.g., Chen et al., 2012; Kieffer, 2012). It is precisely in vocabulary, syntax, and listening comprehension where major differences can be found between native speakers and second-language learners that could impact on reading development (Chen et al., 2012). As readers attempt to comprehend written texts in an L2, differences in their levels of reading comprehension could arise from factors slightly different from those that predict poor performance and individual differences in L1 text comprehension. Geva and Farnia (2012) found exactly this when they studied the predictors of reading comprehension achievement in grade 5 and found a slightly different pattern between native speakers and second-language learners. Besides phonological awareness and vocabulary, predictors common to all children, in the L2 learner group syntax and listening comprehension also added power to the explanation of the variance in reading comprehension.

The aim of this study was to analyze the development of reading comprehension and related abilities in Spanish-immigrant Primary and Secondary school children of different cultural backgrounds, specifically controlling for language and SES, and with Spanish as a common L2. By comparing native Spanish children with those having a Hispanic background, and to others with non-Spanish speaking families, we could analyze separately the impact of language and immigrant status on reading.

The specific aims of our study were: (1) to assess the level of reading comprehension in immigrant students (Hispanic and Non-Hispanic) in comparison to their native peers, when controlling for SES; (2) to compare reading comprehension and related linguistic processes (such as listening comprehension, vocabulary, syntax or monitoring) in these immigrant and native populations at different ages; (3) to analyze the relative contribution of these components, together with age and SES, in explaining individual differences in reading comprehension in immigrant and native reader groups.

We hypothesized that (1) reading comprehension of students with equivalent SES would be similar despite their cultural origin and language, (2) reading comprehension and the related cognitive and linguistic components would grow with age in all groups, but differences should appear in non-Hispanic group with respect to native and Hispanic children in oral language skills, (3) despite similar performance and development, distinctive weights of linguistic and sociocultural aspects in each reading model would show different predictors of reading comprehension in native and immigrant children, with Hispanic and native-Spanish children showing the same predictors of individual differences.

## Materials and Methods

### Participants

We selected 314 participants from 37 different schools of the Southern, Eastern and Northern regions of Spain. There was a total of 157 immigrant-background children (IB, 72 boys and 85 girls) who had both parents born outside Spain, and 157 native children (NA, 66 boys and 91 girls), all of whom were from Spanish-speaking families and participated in a larger nationwide study on reading development. This nationwide study was carried out in 43 public and private schools from several regions of Spain: Basque Country, Murcia, Andalusia, and Canary Islands. It included a total of 4292 children and adolescents aged from 7 to 15 years, enrolled in courses from 2° grade of primary education to 2° grade of secondary education. Within this bigger sample, we took all IB children that participated in the nationwide study and selected a native participant of the same grade and school. Whenever possible, IB children were paired with native participants of the same sex and from the same classroom. Additional inclusion criteria for both groups were a non-verbal IQ higher than 70 or below 130, and no clinical diagnosis or special educational needs.

Within the immigrant sample two groups could be differentiated: 94 *Hispanic* children (HI, 37 boys and 57 girls) who were monolingual-Spanish speakers (two HI children were later excluded from further analyses because they were the only bilinguals in this group), mainly with families from Ecuador (38%), Colombia, and Argentina (18% each); and 61 *Non-Hispanic* children (NH, 33 boys and 28 girls), bilingual speakers who had Spanish as a second language, mainly with families from Morocco (49%) and Romania (21%)—proportions for other European and African nationalities were below 5%. The place of birth for IB participants was Spain for 26.6% of HI and 27.8% of NH (with equivalent proportions of first-/second- generation children in both groups). No significant differences were found between the HI and NH groups in the number of years living in Spain (*MD* = −0.38, *p* = 0.65), the age of arrival in Spain (mean age HI = 3.9 years, NH = 4.2 years), *t*(2, 146) = −0.48, *p* = 0.63, or the number of children that had learned to read in Spain (59.6% of HI children, and 55.7% of NH children), χ^2^(1, 155) = 0.22, *p* = 0.64.

The mean age within the Native group was 9.99 years (*SD* = 2.0), while the mean age of the Hispanic children was 10.03 years (*SD* = 2.1) and the mean age of the Non-Hispanic group was 10.19 years (*SD* = 2.3). There were no significant differences between the native and the immigrant groups in age, gender or non-verbal IQ, *F*(2, 309) = 0.21, *p* = 0.81, η^2^ = 0.001, χ^2^(2, 312) = 3.58, *p* = 0.17, and *F*(2, 309) = 1.75, *p* = 0.18, η*^2^* = 0.01, respectively. Additionally, a SES index was created following Sirin (2005) and Noble et al. (2006). A composite measure of SES was extracted, based on total family income, mean parental educational level, and mean parental occupational status, based on the distribution of the larger reading study from which our participants had been extracted (*N* = 4292). After standardizing the data, a principal components analysis was carried out initially on all the participants with complete data on these three variables (*n* = 3017) and the FactoMineR package of R Language (Husson et al., 2009). Missing cases represented 7.2% for the variable level of total family income, 15.0% for mean parental educational level, and 18.3% for mean parental occupational status. Missing data were replaced using the missMDA package of R Language (Husson and Josse, 2010), employing an iterative PCA (EM) algorithm, with one dimension. The final principal components analysis was computed on the whole original dataset with replaced values. For a three-components solution, only the first component was associated to an eigenvalue higher than 1. All three variables loaded evenly on this component, which explained 82% of total variance. Factor 1 was therefore the only one retained (see the structure coefficients in Appendix). Finally, the coordinate of each individual on the first component of the resulting principal component analysis was included as a global index of socioeconomic status.

Although matched groups of IB and native children of the same schools and grades were selected, there were significant differences in this SES index, *F*(2, 309) = 21.82, *p* < 0.01, η*^2^* = 0.12. This disparity was due to native families having higher income and occupational level than immigrant families, all *ps* < 0.001, despite less years of schooling than HI parents (*p* < 0.01) and a trend to higher than NH (*p* = 0.055). Regarding IB families, HI had higher incomes (*p* < 0.001), years of schooling (*p* < 0.01), and a trend to higher occupational level than NH parents (*p* = 0.06). The group of native children could be considered middle/low-SES, while both immigrant groups could be considered low-SES according to the SES index and compared to the national dataset.

For exploratory purposes, participants were distributed in three age groups: (1) under 9 years old, (2) between 9 and 11 years old, and (3) over 11 years old. The proportion of NA, HI and NH students in each age set was equivalent, χ^2^(4, 312) = 1.01, *p* = 0.91, and there were no significant differences between groups in non-verbal IQ (see Table 1 for a summary of descriptive information).

**TABLE 1:** Means, standard deviations and range for all descriptive measures for native, Hispanic, and non-Hispanic children.

	Native			Hispanic			Non-Hispanic			
Measures	*n*	*M (SD)*	Range	*n*	*M (SD)*	Range	*n*	*M (SD)*	Range	*p*
**Demographic variables**										
Age (in years)	157	9.99 (2.0)	7.0–15.5	94	10.03 (2.1)	6.3–14.2	61	10.19 (2.3)	6.9–15.3	*ns*
Years living in Spain	157	9.92_a_ (2.0)	4.8–15.5	88	5.91_b_ (2.9)	0.0–13.9	60	6.28_b_ (3.1)	0.3–14.6	<0.001
Non-verbal reasoning	157	26.43 (6.0)	14–42	94	25.56 (5.9)	12–42	61	25.41 (5.6)	15–43	*ns*
**Socioeconomic variables**										
Level of income	148	3.55_a_ (1.6)	1–6	94	2.20_b_ (0.9)	1–4	61	1.70_c_ (0.7)	1–3	<0.001*
Years of schooling	157	10.74_b_ (3.5)	3–17	94	11.88_a_ (3.4)	5–23	57	9.40_c_ (4.8)	0–20	<0.01*
Occupational status	145	5.33_a_ (2.2)	1–9	84	6.36_b_ (2.2)	1–9	57	7.05_b_ (1.8)	2–9	<0.001*
SES index	157	−0.38_a_ (1.5)	−3.19 —2.77	94	−1.35_b_ (1.0)	−3.19–1.10	61	−1.40_b_ (1.1)	−3.19 —0.86	<0.001
**Teacher ratings and support**										
Attention ratings	157	3.78 (1.3)	1–6	94	3.63 (1.3)	1–6	61	3.59 (1.4)	1–6	*ns*
Academic support	157	1.15 (0.4)	1–2	94	1.24 (0.4)	1–2	61	1.33 (0.5)	1–2	*ns*
Home stimulation	157	2.79_a_ (0.5)	0–3.9	94	2.51 (0.6)	0–3.5	61	2.39_b_ (0.6)	0–3.5	<0.01
**Reading related measures**										
Reading comprehension	157	58.75 (21.2)	6.2–93.7	94	62.31 (18.9)	2.2–93.7	61	58.10 (19.4)	9.7–93.7	*ns*
Listening comprehension	157	58.18 (20.2)	6.2–93.7	94	52.92 (23.5)	6.2–93.7	61	57.01 (19.3)	6.2–87.5	*ns*
Sentence-picture matching	157	76.56_a_ (12.7)	20.4–100	94	74.38_a_ (12.5)	20.4–100	61	68.87_b_ (14.2)	20.4–92.6	<0.05
Sentence monitoring	157	77.37 (15.3)	13.0–100	94	76.31_a_ (14.1)	30–100	61	70.82_b_ (14.2)	43–98	<0.05
Vocabulary	157	45.29_a_ (9.5)	21–69	94	42.83_b_ (9.2)	19–65	61	33.80_c_ (13.1)	0–64	<0.05
Word reading	157	96.00 (7.5)	46.9–100	94	94.39 (13.3)	9.4–100	61	93.75 (8.6)	62.5–100	*ns*
Non-word reading	157	90.58 (11.5)	35.4–100	94	88.55 (15.4)	13.5–100	61	87.43 (11.4)	50.0–99.0	*ns*

*Letters are used to show significant differences between the groups with different subscripts. Non-verbal IQ (M = 100, SD = 15). Range for raw scores is as follows: non-verbal KBIT raw scores (min 0–max 48), income (min 1–max 6), occupational status (min 1–max 9), SES index (min −3.19–max 3.11), attention in class (min 1–max 6), academic support (min 1/no–max 2/yes), home stimulation (min 0–max 4). All reading related measures refers to accuracy percentage (min 0–max 100), except Vocabulary (<age 8, min 0–max 45; > age 8, min 0–max 82).*

*Refers to levels of significance in non-parametric (Mann-Whitney’s U) analyses.

### Materials

Background measures included Kaufman’s Brief Intelligence Test (K-BIT) (Kaufman, 2000), a sociocultural questionnaire and a teacher questionnaire.

#### Kaufman’s Brief Intelligence Test

We administered the Spanish version of the test that provides two main scores: *Vocabulary*, and Matrices (which was used as *non-verbal IQ* measure). We used the raw scores for both scales. Raw scores range from 0 to 48 for Matrices and from 0 to 82 for the Vocabulary subscale (from 0 to 45 for participants under age 8).

#### Sociocultural Variables Questionnaire

Specifically designed for this study, it was a parental survey on socioeconomic status (family income, parental education, and occupation), languages spoken at home, and a 5-point Likert scale (0–4) of 8 items exploring reading-related practices including reading and writing habits, access to books at home, and the frequency of cultural activities, such as going to exhibitions or bookshops. The *home stimulation* measure was the mean score on the 8 items of the scale.

#### Teachers’ Questionnaire

It was used to record specific diagnoses, academic support (presence or absence of additional school support in any academic domain), and performance in attention skills in class as rated by participants’ teachers in a 6-point Likert scale (1–6). *Attention ratings* and *academic support* measures were the mean of the correspondent responses.

Several experimental tasks were used to evaluate reading comprehension and related abilities. All of them were programmed using E-Prime v2.0 software (Schneider et al., 2002).

#### Word and Non-word Reading

In this task participants read aloud 96 words (e.g., “girlfriend”) and 96 non-words (e.g., “mecanife”) shown in random order in the centre of the computer screen. Data regarding accuracy on reading words and non-words, as well as the response times in both reading options, were obtained. Total scores were calculated as total word and non-word reading accuracy. Internal consistency (alpha) of these tasks was 0.89 in both cases.

#### Reading Comprehension

This task consisted of eight texts that differed in difficulty (four easy and four difficult) and text type (two narrative and two expository within each level of difficulty). Participants read in a self-paced fashion a minimum of two texts and a maximum of four, in a random presentation (one easy-narrative, one easy-expository, one difficult-narrative, one difficult-expository). Each text was followed by four true-or-false literal and inferential questions to evaluate comprehension. The number of texts that participants had to read was determined automatically by their accuracy on the reading comprehension questions. Children in 5th grade or under read two easier texts at the beginning and, if they achieved less than 50% of accuracy, then stopped. If they achieved a higher score, they went on to complete the other two harder texts. Children in 6th grade or above read the two harder texts initially and, if they achieved less than 50% of accuracy, they had to complete the two easier texts. Total score on this task was the total percentage of correct responses to all comprehension answers presented (total accuracy). Internal consistency of this task was 0.90 (Cronbach alpha).

#### Listening Comprehension

The structure of this task was the same as the reading comprehension task, but with orally presented stories. Total scores were calculated as the total accuracy of all comprehension answers. Internal consistency (alpha) was 0.79.

#### Sentence Monitoring

In this task of comprehension monitoring, participants were asked to read silently sentences with suitable vocabulary and easy syntactic structure. Four versions of each sentence were created: (a) semantically incongruent sentences—sentences that contained an adjective semantically incoherent with a noun—, (b) grammatical-gender incongruent sentences—sentences that contained gender disagreement between a noun and an adjective—, (c) grammatical-number disagreement sentences —sentences that contained number disagreement between a noun and an adjective—, and (d) correct sentences—sentences that did not include semantic or grammatical errors. The adjective that made the sentence correct or incorrect varied its position in the sentence, so it was not predictable. The sentences were presented in the middle of the screen and participants had to decide whether the sentence was correct, or it contained an error (correct/incorrect responses, respectively). Each participant read 40 sentences randomly presented (8 semantically incongruent sentences, 8 gender-disagreement sentences, 8 number-disagreement sentences, and 16 correct sentences). Sentences were counterbalanced across participants. Total scores were calculated as the total accuracy of children’s responses. The internal consistency (alpha) was 0.57.

#### Sentence-Picture Matching Syntax Task

This task was adapted from the syntax scale of the PROLEC-R test battery for reading in Spanish (Cuetos et al., 2007). Thirty Spanish sentences were written using easy and suitable vocabulary. The set of sentences ranged in difficulty by increasing their syntactic complexity from simple active sentences (e.g., “the boy reads a book”) to object relative sentences (e.g., “the sheep that the goat chases is white”). For each sentence, four pictures were drawn. One target picture correctly described the meaning of the sentence (e.g., a goat chasing a sheep) and three foil pictures contained wrong descriptions of the sentence (e.g., a sheep chasing a goat). Participants were presented with one sentence in the middle of the screen and four pictures (one target and three foils) randomly located at the corners of the screen. They were instructed to read the sentence and decide which picture correctly depicted its meaning. Sentence types were attributive- and simple-active structures, active sentences containing a negation, passive structures, and sentences containing a focalized object, a split subject, a split object, a subject-subordinate clause or an object-subordinate clause. Sentence order was randomized across participants. Total scores were calculated as the total accuracy of children’s responses. The internal consistency (alpha) was 0.74.

### Procedure

Parental informed consent for inclusion in the study was obtained for all participants. This study was conducted in accordance with the Declaration of Helsinki, and its protocol was approved by the Universidad de La Laguna Research Ethics Committee. All tasks were administered individually in quiet and comfortable areas provided by the schools. Psychology graduates properly trained in these tasks carried out data collection.

The evaluation process began with the administration of the K-BIT, followed by the computerized tasks. To carry out the computerized tasks a desktop PC was used accompanied by a keyboard with two response buttons highlighted in color (the key /L/ on the right of the keyboard, green, and the key /S/ on the left, red), a mouse, and two sets of headphones with integrated microphones (one for the participant and one for the examiner) to listen to the instructions of each test and record the verbal responses of participants when needed. The order of administration of the computerized tasks and the presentation of the assessment items of each task were randomized. All tests had a verbal explanation presented through the headphones and written instructions shown on screen. Each task also included different practice items, in which the examiner could provide help to participants if needed. On average, a 1-h session per participant was needed to complete these computerized tasks. The period between the administration of the K-BIT and the computerized tasks was used to provide the families and teachers with the questionnaires and collect them.

## Results

Aims 1 and 2 (comparison of reading comprehension, decoding, and component skills among groups) were achieved by using a series of ANCOVAs on group membership—Native, Hispanic Immigrants, and non-Hispanic Immigrants—, and age and SES as covariates. Also, accuracy of reading comprehension was compared for these three groups to the results from a large sample of native schoolchildren from different areas of Spain.

For Aim 3 (models of reading comprehension in the different groups), we carried out a regression analysis with age, non-verbal reasoning, SES, group membership, and the different components, as predictors of reading comprehension.

### Preliminary Analyses

Twenty-nine participants had some missing data, compromising 1.2% of the total data set. Missing data appeared in reading and listening comprehension (1.9 and 0.3%, respectively), sentence monitoring (0.6%), sentence-picture matching (1.0%), academic support (0.6%), and attention ratings (5.4%). We ran a multiple imputation analysis to estimate missing data using the SPSS v.20 Multiple Imputation procedure with five iterations, based on linear regression for the test measures and logistic regression for teacher ratings and support measures.

### Descriptive and Comparative Analyses

Mean accuracy reading comprehension scores were 58.7 (*SD* = 21.2), 62.3 (*SD* = 18.9), and 58.1 (*SD* = 19.4) for Native, Hispanic and non-Hispanic children, respectively (see Table 1). Native participants in the larger study mentioned above had an overall mean score of 63.3 (*SD* = 20.9). The Native group had a total of 36.9% of participants in the lowest quartile of the larger population sample. In the case of the Hispanic and non-Hispanic immigrants these proportions were of 26.3 and 34.6%, respectively. The proportions of children in the highest quartile were 17.0, 12.5, and 10.9% for the NA, HI, and NH groups, respectively. In the native population of the larger study, the proportions of children in the lowest and highest quartile were 27.0 and 21.0%, respectively. Taking reading comprehension scores of the native population of the larger study as a standard, we can determine that our native and immigrant background groups showed a lower performance (η^2^ = 0.22, and 0.15, respectively). NA and NH groups performed similarly low, while HI participants showed no mean differences compared with the larger study group, but where underrepresented in the highest quartile (see Appendix for a summary of this analyses).

A series of ANCOVAs exploring the impact of age, SES, and origin were carried out on all main variables (see Table 2). All variables—reading comprehension, listening comprehension, sentence monitoring, sentence-picture matching, vocabulary, and word/non-word reading—showed a significant B parameter of age (*p*s < 0.0001, see Table 2). A significant B parameter of SES was also found for all of them (*p*s < 0.024), except for reading comprehension (*p* = 0.07) and word/non-word reading (*p*s > 0.946).

**TABLE 2:** Analysis of covariance summary.

Measures	Sum of squares	df	*F*	*p*
**Reading comprehension**				
SES	1,148	1	3.24	0.073
Age	10,273	1	29.02	<0.0001
Origin	1,541	2	2.18	0.115
Age × Origin	243	2	0.34	0.709
**Listening comprehension**				
SES	2,014	1	5.12	0.024
Age	12,430	1	31.60	<0.0001
Origin	978	2	1.24	0.290
Age × Origin	1,216	2	1.55	0.215
**Sentence-picture matching**				
SES	855	1	5.95	0.015
Age	2,725	1	18.95	<0.0001
Origin	1,746	2	6.07	0.003
Age × Origin	107	2	0.37	0.690
**Sentence monitoring**				
SES	2,503	1	13.59	<0.001
Age	6,634	1	36.01	<0.0001
Origin	1,340	2	3.64	0.027
Age × Origin	1,034	2	2.81	0.062
**Vocabulary**				
SES	631	1	13.33	<0.001
Age	16,418	1	346.93	<0.0001
Origin	5,193	2	54.87	<0.0001
Age × Origin	62	2	0.66	0.517
**Word reading**				
SES	0	1	0.00	0.997
Age	3,069	1	26.57	<0.0001
Origin	279	2	1.21	0.300
Age × Origin	47	2	0.20	0.817
**Non-word reading**				
SES	1	1	0.00	0.946
Age	7,131	1	40.83	<0.0001
Origin	517	2	1.48	0.229
Age × Origin	134	2	0.38	0.682

A non-significant relationship of origin was found for reading comprehension (*p* = 0.12) or listening comprehension (*p* = 0.29). There were also no significant interactions between age and origin (*p* = 0.71 and *p* = 0.21, respectively). We found the same for the word/non-word reading measures, which showed no significant differences for origin (*p*s > 0.23) and no significant interactions between age and origin (*p*s > 0.68).

Analyses of sentence-picture matching, sentence monitoring, and vocabulary did show significant differences for origin (*p*s *< 0*.027). No significant interaction between age and origin was found in these variables (*p*s > 0.06). In sentence-picture matching, Non-Hispanic children performed more poorly than native, *p* = 0.002, and Hispanic children, *p* = 0.040. Similarly, in vocabulary, the NH group performed below the HI and the NA groups (*p*s < 0.0001). Surprisingly, the HI group also performed marginally below the native children (*p = 0*.045). In sentence monitoring, the NH group had poorer results than the HI group (*p* = 0.03) and marginally than the native group (*p* = 0.06).

Two additional ANCOVAs were carried out across groups on word and non-word reading accuracy, with SES as a covariate. Again, age turned out to be a significant predictor of decoding (*p*s < 0.0001), but there were no significant B parameters for origin (*p*s > 0.23).

Our sample included both children born in Spain and abroad. We compared performance in the HI and NH groups between children with different birthplace with a series of *t-*tests. There were no differences between first/second generation NH children in any of the measures. However, in the case of HI students, we did find differences in reading comprehension and vocabulary, but they disappeared once age was controlled for. HI first-generation children were older than their second-generation peers (*MD* = 2.56, *p* < 0.001). Thus, a series of ANCOVA with age as a covariate were performed in HI students. Age was a significant predictor in all analyses (*ps* < 0.008), but there were no significant differences between first/second generation HI children on any of the measures (*p*s > 0.348).

### Reading Models

Bivariate correlations for the three origin groups, NA, HI, and NH are presented in Table 3. In order to evaluate the reading comprehension model and the variables that best predict it in the three groups of origin, we performed a regression analysis for reading comprehension, with linguistic, cognitive, cultural origin, SES, non-verbal reasoning, and contextual variables as predictors (see Table 4). Considering that standardized regression weights can incorrectly partition variance when predictors are correlated, as in the present study, we also used dominance analysis to complement these multiple regression analyses (Budescu, 1993), using the *relaimpo* R statistical package (Groemping, 2006; R Core Team, 2013). This procedure allows one to assess the unique contribution of each variable to the regression model in the presence of the other predictors (Azen and Budescu, 2003; Feldman, 2005; Groemping, 2006).

**TABLE 3:** Correlations among all measures for Native, Hispanic, and Non-Hispanic children.

Measures	1	2	3	4	5	6	7	8	9	10	11	12
**Native *(n = 157)***												
1. Reading comp.	1											
2. Word reading	0.25**	1										
3. Non-word reading	0.25**	0.86***	1									
4. Listening comp.	0.28***	0.27**	0.22**	1								
5. S-P matching	0.19*	0.20*	0.24**	0.24**	1							
6. S Monitoring	0.41***	0.42***	0.41***	0.45***	0.42***	1						
7. Vocabulary	0.37***	0.47***	0.51***	0.41***	0.42***	0.54***	1					
8. Attention ratings	0.23**	0.22**	0.23**	0.17*	0.22**	0.19*	0.19*	1				
9. Academic support	–0.12	−0.34***	−0.36***	–0.16	–0.16	–0.12	–0.16	−0.31***	1			
10. Home stimulation	0.23**	0.20*	0.17*	0.02	0.17*	0.25**	0.35***	0.14	–0.10	1		
11. Age in months	0.33***	0.39***	0.43***	0.39***	0.21*	0.45***	0.79***	–0.06	0.02	0.11	1	
12. N-verbal reasoning	0.42***	0.41***	0.43***	0.43***	0.35***	0.53***	0.76***	0.24**	–0.09	0.32***	0.63***	1
13. SES	0.16	0.12	0.09	0.30***	0.19*	0.23**	0.29***	0.23**	–0.10	0.29***	0.08	0.35***
**Hispanic (*n* = 94)**												
1. Reading Comp.	1											
2. Word Reading	–0.04	1										
3. Non-word reading	0.07	0.93***	1									
4. Listening Comp.	0.11	–0.11	–0.10	1								
5. S-P Matching	0.20	0.25*	0.38***	0.06	1							
6. S Monitoring	0.24*	0.28*	0.41***	0.02	0.48***	1						
7. Vocabulary	0.38***	0.36**	0.47***	0.05	0.50***	0.50***	1					
8. Attention ratings	0.05	0.24*	0.30**	–0.14	0.27*	0.27*	0.16	1				
9. Academic support	–0.12	–0.17	–0.21	0.16	–0.16	−0.39***	–0.16	−0.34**	1			
10. Home stimulation	0.18	–0.15	–0.11	0.12	–0.02	0.07	0.21	0.10	–0.06	1		
11. Age in months	0.32**	0.22	0.25*	0.21	0.24*	0.19	0.71***	–0.20	0.12	0.00	1	
12. N-verbal reasoning	0.45***	0.23*	0.33**	0.01	0.32**	0.49***	0.76***	0.28*	0.11	0.13	0.58***	1
13. SES	0.02	0.02	0.05	0.01	0.10	0.30**	0.23*	0.16	0.34**	0.14	0.09	0.29*
**Non-Hispanic (*n* = 61)**												
1. Reading comp.	1											
2. Word reading	0.27*	1										
3. Non-word reading	0.29*	0.90***	1									
4. Listening comp.	–0.08	0.13	0.22	1								
5. S-P matching	0.23	0.55***	0.44***	0.14	1							
6. S Monitoring	0.27*	0.53***	0.52***	0.19	0.59***	1						
7. Vocabulary	0.33*	0.40**	0.36**	0.22	0.58***	0.56***	1					
8. Attention ratings	–0.07	0.15	0.14	0.05	0.38**	0.30*	0.28*	1				
9. Academic support	0.07	–0.14	–0.11	0.02	–0.23	–0.21	–0.16	−0.46***	1			
10. Home stimulation	0.10	0.10	0.01	0.01	0.07	0.05	0.15	0.02	−0.31*	1		
11. Age in months	0.28*	0.46***	0.45***	0.29*	0.33*	0.34*	0.72***	0.17	0.12	0.10	1	
12. N-verbal reasoning	0.26	0.37**	0.35**	0.06	0.45***	0.43**	0.62***	0.26	–0.14	0.31*	0.66***	1
13. SES	–0.06	0.33*	0.25	–0.16	0.06	0.02	–0.13	–0.04	–0.12	0.20	–0.02	0.17

*p < 0.05; **p < 0.01; ***p < 0.001.

**TABLE 4:** Predictors of reading comprehension in the full sample including two dummy variables for immigrant status (HI and NH).

Variable	*B*	*SE B*	*t*	95% CI
Constant	0.000	14.77	1.23	[−10.91, 47.26]
Age	0.093	0.93	0.94	[−0.96, 2.70]
Non-verbal reasoning	0.233	0.30	2.62**	[0.20, 1.38]
SES	–0.040	0.88	–0.62	[−2.29, 1.19]
Word reading	–0.172	0.25	–1.39	[−0.84, 0.14]
Non-word reading	0.132	0.20	1.01	[−0.19, 0.60]
Listening comprehension	0.030	0.06	0.49	[−0.08, 0.14]
Sentence-picture matching	–0.004	0.11	–0.06	[−0.22, 0.20]
Sentence monitoring	0.171	0.10	2.30*	[0.03, 0.42]
Vocabulary	0.022	0.22	0.18	[−0.39, 0.47]
Attention ratings	0.021	0.96	0.33	[−1.57, 2.20]
Academic support	–0.010	3.08	–0.16	[−6.54, 5.58]
Home stimulation	0.058	0.23	0.92	[−0.25, 0.68]
Origin Hispanic	0.163	2.70	1.50	[−1.27, 9.38]
Origin non-Hispanic	1.969	3.58	1.12	[−3.05, 11.04]
*R* ^2^	0.211			
*F* for change in *R*^2^	4.906***			

*N = 312. CI, confidence interval.*

*p < 0.05; **p < 0.01; ***p < 0.001.

*R* for the overall regression model was significantly different from zero, *F*(14, 257) = 4.91, *p* < 0.0001, and *R*2 = 0.21. Of the predictors, the only individual variables with a coefficient significantly different from zero were sentence monitoring (*p* = 0.03), and non-verbal reasoning (*p* = 0.009). Group of origin coefficients were not significantly different from zero, and neither were the coefficients of any interactions involving group.

Dominance analysis showed that age, non-verbal reasoning, and SES, jointly explained 16.9% of the variance. The relative importance metric LMG (Groemping, 2006) confirmed that, of the other predictors (controlling for age, non-verbal reasoning, and SES), sentence monitoring explained most of the remaining 4.18% (2.0%), at a great distance from the next contributing predictor (origin—0.8%) (see Table 5).

**TABLE 5:** Relative importance metrics in the dominance analysis of regressors, controlling age, non-verbal reasoning, and SES.

Variable	% variance	95% CI
Word reading	0.41	[0.05, 2.12]
Non-word reading	0.23	[0.05, 1.50]
Listening comprehension	0.16	[0.01, 1.66]
Sentence-picture matching	0.10	[0.04, 1.66]
Sentence monitoring	1.98	[0.16, 5.89]
Vocabulary	0.10	[0.05, 1.50]
Attention ratings	0.07	[0.01, 2.15]
Academic support	0.07	[0.02, 1.80]
Home stimulation	0.25	[0.01, 2.62]
Origin	0.80	[0.09, 4.08]
*Total variance partitioned*	4.18	

*N = 312. CI, confidence interval.*

## Discussion

### Differences Among Groups

Previous studies on reading comprehension in immigrant students have pointed to the importance of contextual and socioeconomic factors for explaining differences in their performance with respect to native students (e.g., Lesaux, 2012; Singh, 2013; Melby-Lervåg and Lervåg, 2014; Marx et al., 2015). In our study, we have attempted to exert a maximal control over socioeconomic and contextual factors by closely controlling for parental SES and schools. For each immigrant participant, a child from his or her school and age group was selected. In addition, we specifically developed a SES index score for each participant based on individual parental occupational status, family income, and educational attainment, which was then statistically controlled for. We also separated immigrant groups according to whether they shared the majority language (Spanish) or not.

With this procedure, the differences between immigrant and native groups in reading comprehension were non-significant, as we had predicted. We had hypothesized that there would be differences on oral language skills only between the native and the non-Hispanic group. This hypothesis was mostly confirmed. Between-group analyses showed differences in vocabulary, comprehension monitoring, and specific syntactic processes: non-Spanish-speaking immigrants performed more poorly than native and Spanish-speaking immigrant children. The results obtained in this study are consistent with previous research which mostly shows significant differences in vocabulary and syntax between immigrant students who learn to read in a L2 (e.g., Droop and Verhoeven, 2003; Leikin et al., 2010; Lervåg and Aukrust, 2010; Burgoyne et al., 2011; Geva and Farnia, 2012; Rodriguez et al., 2012) and native and immigrant students who do so in their L1 (e.g., Navarro and Huguet, 2005; Proctor et al., 2012; Raudszus et al., 2018).

There were no differences on syntax between Hispanic immigrants and native children as predicted. We did find differences in the vocabulary task, in which Hispanic children performed better than non-Hispanics, but not as well as native-Spanish children. Both groups speak different varieties of Spanish, with different degrees of differentiation in vocabulary, morphology, syntax, and prosody that could explain this finding, as shown in normalization studies of vocabulary tests carried out in Spanish-speaking pediatric population (Olabarrieta-Landa et al., 2017; Rivera and Arango-Lasprilla, 2017).

Apparently, these differences are not sufficient to grossly affect reading comprehension. Whichever mechanisms immigrant children are using to compensate for differences in syntax and/or vocabulary seem appropriate to overcome potential limitations in reading. This compensation could also be present in listening comprehension, which would explain the lack of differences also on that task. The amount and quality of input might not be sufficient to achieve native levels of oral competence, but enough to attain adequate levels of oral and written text comprehension. An alternative explanation is of course a lack of sensitivity of our listening and reading comprehension tasks to subtle differences, or that they were not demanding enough. However, the lack of floor or ceiling effects on either task does not support this explanation.

The differences between non-Hispanic and native-Spanish children in comprehension monitoring are somewhat surprising. However, this error-detection task requires a great deal of linguistic knowledge. The kind of errors included are mostly related to syntactic and lexical anomalies and could thus be greatly influenced by L1 differences, as was found by Raudszus et al. (2018) when analyzing lexical quality and executive control as predictors of first and second language children’s reading comprehension.

The analysis of differences in reading comprehension among groups adds some data to conflicting results in the literature. Although many previous studies did use control children from the same classes, they did not measure individual SES data as we did. An exception of studies that controlled for individual SES and did find differences is Kigel et al. (2015). Like us, they obtained individual data on parental occupational status, together with a measure of cultural capital in the family (extracted from the number of books available at home). They found that immigrant status was still a predictor of reading comprehension differences after accounting for SES. They did, however, have a more varied group of participants in terms of decoding skills, while our groups were matched on word and non-word reading abilities.

One of the more relevant contributions of our study is the dissociation of language and immigrant status by including a group of children of immigrant background with the same L1 as the majority-native population. Our data suggest that language differences are the greatest contributor to differences in immigrant groups. Of course, additional cultural variables should not be ruled out when considering overall academic performance. Even in our case, Hispanic and non-Hispanic (mostly Moroccan and Romanian) immigrant families differ in many ways other than language, since they come from different cultural backgrounds.

Based on the SES Index obtained from the nationwide sample, all our participants, natives, and especially IB children, can be considered to be in the low-SES range of our Spanish sample. In Cummins’s (2000) view, children from low-socioeconomic background should have more difficulty accessing reading in a second language, as a consequence of less-context-independent language at home. However, it could be that our control group has in itself already poorer home contexts and is already performing in the low range. Although our data is limited in this sense, our native sample has a higher proportion of individuals in the lowest quartile of reading comprehension scores, when compared to a larger population study of native Spanish children. This suggests that immigrants and low-SES native children might be affected by the same negative contextual factors.

### Predictors and Components of Reading Comprehension

A main predictive effect of age, with a complete lack of interaction with immigrant status, was found for all measures, as expected. Improved performance with age supports the validity of the tasks and is consistent with our predictions.

Our results are in accordance with studies that find no increase in differences between native and immigrant pupils across ages. Baumert et al. (2012) actually found a decrease in differences in reading comprehension during school years in a longitudinal study, although theirs was a heterogeneous immigrant group. Kigel et al. (2015) found that, although immigrant status did predict different baselines in reading comprehension development, it did not explain the slope of improvement over ages, indicating similar growth patterns.

A joint regression analysis on all the groups was computed to test for predictors of differences in reading comprehension. Most of the variability was explained by age, SES, and non-verbal reasoning. Of the remaining variables, only comprehension monitoring was an individual significant predictor, explaining a small proportion of variance, but clearly more than any other variable. Group of origin did not significantly contribute to explain reading comprehension variability, and no interaction involving this variable was significant. Our hypothesis that different groups would have different developmental pathways or predictors was not supported by the data. On the contrary, this regression analysis adds to the conclusion that, in reading comprehension, these groups are more similar than different.

It could also be the case that factors other than language status should be considered. Home literacy-related experiences have been pointed out as relevant in reading development of Latino children in the United States (see Arzubiaga et al., 2002, for example). Background knowledge could also be a specific factor that was not accounted for in this study and could differentiate amongst Hispanic students (August et al., 2006). As we have already indicated above, cultural factors may have an even larger weight in the non-Hispanic group. Around 50% of this group was composed of children of Moroccan families, another 20% of Romanian families, and the rest of different nationalities. Due to sample size, the actual family language was not included in the regression analyses. It could be that various groups are performing differently, both due to more or less cultural similarities between home literacy-practices (Arzubiaga et al., 2002), and also linguistic similarity between their L1 and Spanish (Melby-Lervåg and Lervåg, 2014). The use of Spanish at home could also vary from family to family.

### Limitations and Future Directions

Among the limitations of this study, we should consider the possible influence of the use of experimental tests. True-or-false and multiple-choice tests—like those used here—tend to show smaller differences between immigrant and non-immigrant groups (Melby-Lervåg and Lervåg, 2014). In addition, one of our tasks, sentence monitoring, had a relatively low reliability. This poorer performance of the task probably derives from the fact that it includes very different types of errors (gender, number, and semantic disagreements).

The lack of information about proficiency in oral Spanish of the immigrant and even the native children is also a restriction in our data set. In this sense, Proctor et al. (2012), found that bilinguals proficient in English performed like their native peers, while both groups outperformed the group of bilingual children that were considered as English language learners by their teachers. The lack of this measure in our NH group may have attenuated some differences in performance with their native and Hispanic peers, as variability in oral Spanish in this group may be very great. Additionally, the non-random nature of the sampling and the low sample size, along with the lack of bilingual natives and bilingual Hispanic children among the analyzed sample may limit our conclusions and hamper their generalization to other regions where bilingualism is the norm.

The results might also have been different if higher-SES students and schools had been included. Nevertheless, we suggest that the lack of differences in our study could be related to a generally low performance of a generally low-SES sample. In any case, these results, with a low-SES group of participants that teases apart the influence of language and immigrant status, contribute to a somewhat contradictory literature about reading comprehension of immigrant pupils. New studies following this procedure are needed. Future research should focus not only on the different languages that immigrant children speak and their proficiency in the language used at school, but also in the relationship that their economic, social and cultural status may have with their reading comprehension performance. Random sampling and a bigger sample, including high-SES students and bilingual natives, would be also desirable to improve the representativeness of the findings. Finally, in order to better track developmental changes in reading comprehension and related cognitive and linguistic processes across native and immigrant children and adolescents, longitudinal studies would be highly recommended.

## Conclusion

The main finding of this study emphasizes that, once SES is controlled for, reading comprehension achievement of immigrant students (both Hispanic and Non-Hispanic) is equivalent to their native Spanish classmates. The study also partially separates linguistic differences from cultural factors. Even if similar levels of reading comprehension are attained, children with different L1s show lower performance on oral language tasks. These skills are important and could impact overall academic achievement in other ways. Also, even when language is apparently very close, as in the case of Hispanic and native students, subtle differences could remain in areas such as vocabulary. Without excluding the influence of cultural factors, this study highlights the importance of adequate L2 oral language skills. It emphasizes current measures undertaken in the Spanish educational system, where children with poor Spanish are offered inclusion in special support units and transferred to mainstream classes when they reach adequate levels of oral language. Although most immigrant children have a sufficient level of Spanish to be in a regular classroom, when they had language difficulties it is crucial for their academic development to receive regular support provided for children with learning difficulties, which includes support teachers, pull-out lessons, and curricular adaptations.

In this sense, our results should not be seen as an argument against providing additional academic support to immigrant students that perform similarly to their native peers. Quite to the contrary, the results obtained in the present study converge with the perspectives which claim that socioeconomically disadvantaged students are a major risk group, no matter if they are native or from an immigrant background.

## Data Availability Statement

The raw data supporting the conclusions of this article will be made available by the authors, without undue reservation.

## Ethics Statement

The studies involving human participants were reviewed and approved by the Universidad de La Laguna Research Ethics Committee. Written informed consent to participate in this study was provided by the participants’ legal guardian/next of kin.

## Author Contributions

JI-A and DS: conceptualization, writing—original draft preparation, and investigation. JI-A, JH-C, JD, AE, PM, MB, LF, and DS: methodology, writing—review, and editing. JI-A, JH-C, AE, and DS: formal analysis. JD, PM, MB, LF, and DS: resources. JI-A and JH-C: data curation. MB, LF, and DS: supervision, project administration, and funding acquisition. All authors have read and agreed to the published version of the manuscript.

## Conflict of Interest

The authors declare that the research was conducted in the absence of any commercial or financial relationships that could be construed as a potential conflict of interest.

## Publisher’s Note

All claims expressed in this article are solely those of the authors and do not necessarily represent those of their affiliated organizations, or those of the publisher, the editors and the reviewers. Any product that may be evaluated in this article, or claim that may be made by its manufacturer, is not guaranteed or endorsed by the publisher.

## Appendix

**TABLE A1:** Structure coefficients of the principal components analysis for the SES Index.

	Component 1	Component 2	Component 3
Variance	2.47	0.30	0.23
% of Variance	82.46	10.02	7.52
Cumulative % of variance	82.46	92.48	100.00

**TABLE A2:** Correlations of variables with the principal components for the SES Index.

	Component 1	Component 2	Component 3
Level of income	0.896	0.426	0.127
Years of schooling	0.922	–0.086	–0.378
Occupational status	0.906	–0.334	0.259

**TABLE A3:** Reading comprehension accuracy and percent quartile distribution.

	Native-NW	Native	Hispanic	Non-Hispanic
Mean accuracy (SD)	63.3 (20.9)	58.7 (21.2)	62.3 (18.9)	58.1 (19.4)
Q1—Lowest	27.0	36.9	26.3	34.6
Q2	29.4	23.4	31.3	23.6
Q3	22.6	22.7	30.0	30.9
Q4—Highest	21.0	17.0	12.5	10.9

*Reading comprehension accuracy percentage (min 0–max 100). Native-NW = nationwide native sample with complete data on reading comprehension accuracy (n = 3,155).*
